# Incidence of neurocutaneous melanosis in Japanese pediatric patients with congenital melanocytic nevi

**DOI:** 10.1038/s41598-023-43829-w

**Published:** 2023-09-30

**Authors:** Miyuki Takiya, Yasutaka Fushimi, Michiharu Sakamoto, Takeshi Yoshida, Kentaro Ueno, Satoshi Nakajima, Akihiko Sakata, Sachi Okuchi, Sayo Otani, Hiroshi Tagawa, Naoki Morimoto, Yuji Nakamoto

**Affiliations:** 1 Department of Diagnostic Imaging and Nuclear Medicine, Graduate School of Medicine, Kyoto University, 54 Shogoin Kawaharacho, Sakyoku, Kyoto 6068507 Japan; 2 Department of Plastic and Reconstructive Surgery, Graduate School of Medicine, Kyoto University, Kyoto, 606-8507 Japan; 3 Department of Pediatrics, Graduate School of Medicine, Kyoto University, Kyoto, 606-8507 Japan; 4 Department of Biomedical Statistics and Bioinformatics, Graduate School of Medicine, Kyoto University, Kyoto, 606-8507 Japan

**Keywords:** Paediatric research, Diseases, Skin diseases

## Abstract

Neurocutaneous melanosis (NCM) is a rare, non-hereditary neurocutaneous disorder characterized by excessive melanocytic proliferation in the skin and central nervous system. As no major studies have covered the incidence of NCM among Japanese patients with congenital melanocytic nevi (CMN), we prospectively investigated the incidence of NCM among Japanese patients who underwent initial treatment for CMN. The relationship of CMN and NCM was also investigated. Japanese pediatric patients with CMN under 1 year of age were included between January 2020 and November 2022, and all patients underwent brain MRI to check for NCM in this study. NCM lesions were most frequently seen in the amygdala, followed by the cerebellum, brainstem, and cerebral hemispheres. NCM was diagnosed on brain MRI in 31.6% of the 38 patients with CMN and in 25.0% of patients with no prior examination or treatment. Distribution and size of CMN, number of satellite nevi, rugosity and nodules were strongly associated with the existence of NCM, and these findings may guide a future registry study with a large cohort of CMN patients.

## Introduction

Neurocutaneous melanosis (NCM) is a rare, non-hereditary neurocutaneous disorder characterized by excessive proliferation of melanocytes in the skin and central nervous system (CNS)^1,2^. NCM refers to the concurrence of congenital melanocytic nevi (CMN) and melanosis in the CNS. Postzygotic mosaic mutation in the embryonic precursor cells of melanocytes in the ectoderm causes congenital melanocytic nevi on the skin, and can also be found in the CNS^3,4^.

Since most patients with NCM are asymptomatic, magnetic resonance imaging (MRI) of the brain is required to confirm the diagnosis of NCM in current clinical practices. Characteristic MRI findings in patients with NCM have been reported, such as regions of abnormal hyperintensity on T1-weighted imaging (T1WI) in the brain parenchyma and/or diffuse leptomeningeal lesions that are considered to reflect melanin deposition or melanocytic proliferation^5–11^.

Some risk factors for NCM have been described in previous reports. An internet-based registry of patients with CMN showed that a large number of satellite nevi represents the most important risk factor for NCM, and that most cases of NCM were symptomatic^12^. A large registry cohort of pediatric and adult patients with CMN showed increasing numbers of satellite lesions and larger diameters of the CMN are associated with malignant melanoma and NCM^13^. However, the overall incidence of NCM has differed among studies, probably due to different criteria and differences in the patient population enrolled^3,5,7,14–16^. In addition, studies into the relationship between CMN and NCM have mainly focused on patients in western countries^5,14–18^.

No major studies have covered the incidence of NCM among Japanese patients with CMN. The purpose of the present study was therefore to reveal the incidence of patients with NCM among Japanese patients who undergo initial treatment for CMN. We also investigated the relationship between CMN and NCM in our cohort.

## Materials and methods

### Patients

**Figure 1:**
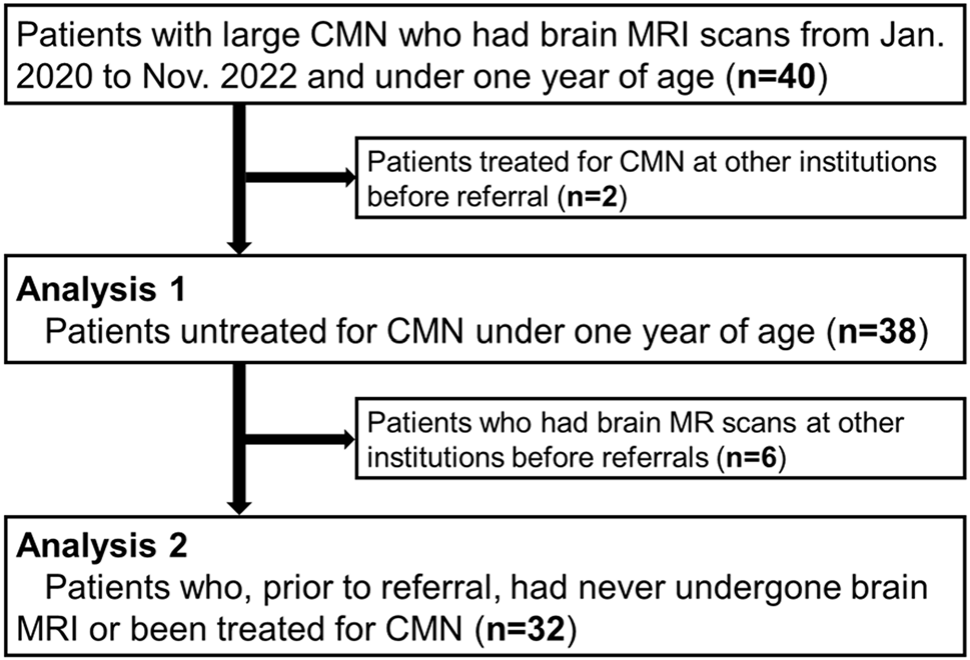
Flowchart of enrollment is shown.

This prospective study was performed in accordance with the Declaration of Helsinki and was approved by Kyoto University Graduate School and Faculty of Medicine, Ethics Committee. A total of 40 pediatric patients under 1 year old [20 males, 20 females; average age, 5.3 months (median age, 4 months; range, 3–12 months)] with CMN, who were referred to our institute from all over Japan between January 2020 and November 2022, were prospectively included in this study (Fig. 1). Written informed consent was obtained from the parents of all patients. The upper age limit was defined because the development of myelination tends to obscure hyperintensity lesions on T1WI. All patients underwent brain MRI as screening for NCM. We defined patients with NCM in this study as those patients who showed a hyperintense lesion in the brain parenchyma and/or abnormal leptomeningeal enhancement on T1WI.

The exclusion criteria were as follows: motion artifacts during MRI; and patients with pathologies other than melanocytic nevi; and treatment for CMN at another institute.

#### Analysis 1

This analysis was conducted to determine the relationship between characteristics of CMN and NCM, and included all patients with untreated CMN. The incidence of NCM was also calculated.

#### Analysis 2

Since no registry study of CMN had been performed in Japan, the incidence of NCM among patients with CMN was unknown. Prior to any future registry study, we investigated the incidence of NCM among treatment-naive patients for CMN. We focused on patients who had never undergone brain MRI and had not been treated for CMN before referral to our institute (no previous MRI examination and treatment group).

### Evaluation of neurological manifestations

All patients were evaluated for neurological symptoms by a board-certified pediatric neurologist (T.Y., with 16 years of experience in pediatric neurology).

### Evaluation of CMN

The size, anatomical localization and characteristic features of CMN were recorded and evaluated by a board-certified plastic surgeon (M.S., 23 years of experience in plastic surgery) for all patients, based on the categorization proposed by Krengel et al.^19^.

The size of the CMN was categorized by calculating the projected adult size as follows: G2, > 60 cm; G1, 40–60 cm; L2, 30–40 cm; L1, 20–30 cm; M2, 10–20 cm; and M1, 1.5–10 cm.

Anatomical localizations were classified as follows: face, scalp, neck, shoulder, upper back, middle back, lower back, breast/chest, abdomen, flank, gluteal lesion, genital lesion, upper arm, forearm, hand, thigh, lower leg, and foot.

Anatomical distributions of CMNs on the skin surface of patients were categorized as follows: bonce (head and facial region), bolero (mainly on the upper back, including the neck), back (usually in a round shape and not involving the buttocks or shoulders), bathing trunk (mainly involving the genital region and buttocks, excluding the shoulders and neck), breast/belly (only distributed on the chest or abdomen and showing no overlap with bolero or bathing trunk), body extremity (only located on the extremities and excluding the shoulders and genital region) and body (a pattern combining bolero and bathing trunk patterns, and affecting almost the entire body)^20,21^.

Morphological characteristics were used to characterize CMN into grades 1–3 in terms of color, heterogeneity, surface rugosity, and the presence of dermal or subcutaneous nodules^20,21^. Hypertrichosis was not evaluated in this study because the definitions remain ambiguous, especially for lesions around the head.

The number of satellite nevi was categorized into grades 0–3 as follows: grade 0, no nevi; grade 1, 1–19 nevi; grade 2, 20–50 nevi; and grade 3, > 50 nevi.

### MRI

All patients underwent MRI of the brain using a 3-T system (MAGNETOM Prisma or Skyra; Siemens Healthineers, Erlangen, Germany) under sedation conducted by pediatricians. Scans included 3D T1WI, 3D T2-weighted imaging (T2WI), susceptibility-weighted imaging (SWI), and diffusion-weighted imaging (DWI). Imaging parameters for 3D T1WI were: T1-SPACE; repetition time (TR), 600 ms; echo time (TE), 9 ms; flip angle (FA), 120°; field of view (FOV), 220 × 256 mm; in-plane resolution, 1 × 1 mm; slice thickness, 1 mm; acceleration factor, Wave-CAIPI 9 × ; and scan time, 1 min 49 s. Imaging parameters for 3D T2WI were: T-SPACE; TR, 4000 ms; TE 299 ms; FA, 120°; FOV, 220 × 256 mm; in-plane resolution, 1 × 1 mm; slice thickness, 1 mm; acceleration factor, Wave-CAIPI 9 × ; and scan time, 1 min 44 s. Imaging parameters for SWI were: gradient echo; TR, 39 ms; TE, 20 ms; FA, 15°; FOV 175 × 200 mm; in-plane resolution 0.6 × 0.6 mm; slice thickness, 1.5 mm; acceleration factor, generalized autocalibrating partial parallel acquisition (GRAPPA) 2 × ; and scan time, 4 min 51 s. Imaging parameters for DWI were: TR, 5700 ms; TE 77 ms; FA, 90°; FOV, 220 × 220 mm; in-plane resolution, 1 × 1 mm; slice thickness, 3 mm; acceleration factor, GRAPPA 2 × ; and scan time, 58 s.

### Evaluation of NCM

NCM was defined in this study as any abnormal hyperintensity lesion on T1WI. Locations of abnormal hyperintensities on T1WI were recorded by one board-certified radiologist (M.T., 7 years of experience in neuroradiology) and approved by another board-certified radiologist (Y.F., 24 years of experience in neuroradiology). Abnormalities noted on other imaging sequences were also recorded.

### Statistical analysis

Relationships between NCM and characteristics of CMN were evaluated using Fisher’s exact test. Values of p < 0.05 were considered statistically significant. JMP Pro software (version 16.2.0; SAS Institute, Cary, NC, USA) was used for all statistical analyses.

## Results

### Patients

Forty patients with CMN under one year old were included in this study. No patients were excluded due to motion artifacts during MRI. No family history of CMN or melanosis was identified. Two female patients treated for CMN before being referred to our institution were excluded, so a final total of 38 pediatric patients (20 males, 18 females) were included for Analysis 1. In the next step, 32 patients (17 males, 15 females) with CMN who had not undergone any MRI of the brain before referral were included for Analysis 2.

Only one female patient with bilateral amygdala lesions (right-side predominance) showed repetitive epileptic seizures and mild electroencephalographic examination abnormality during the course of her disease.

Pathological diagnosis for CMN was performed for all patients in our institution, and all specimens were confirmed to be negative for malignancy.

### Diagnosis of NCM

**Figure 2:**
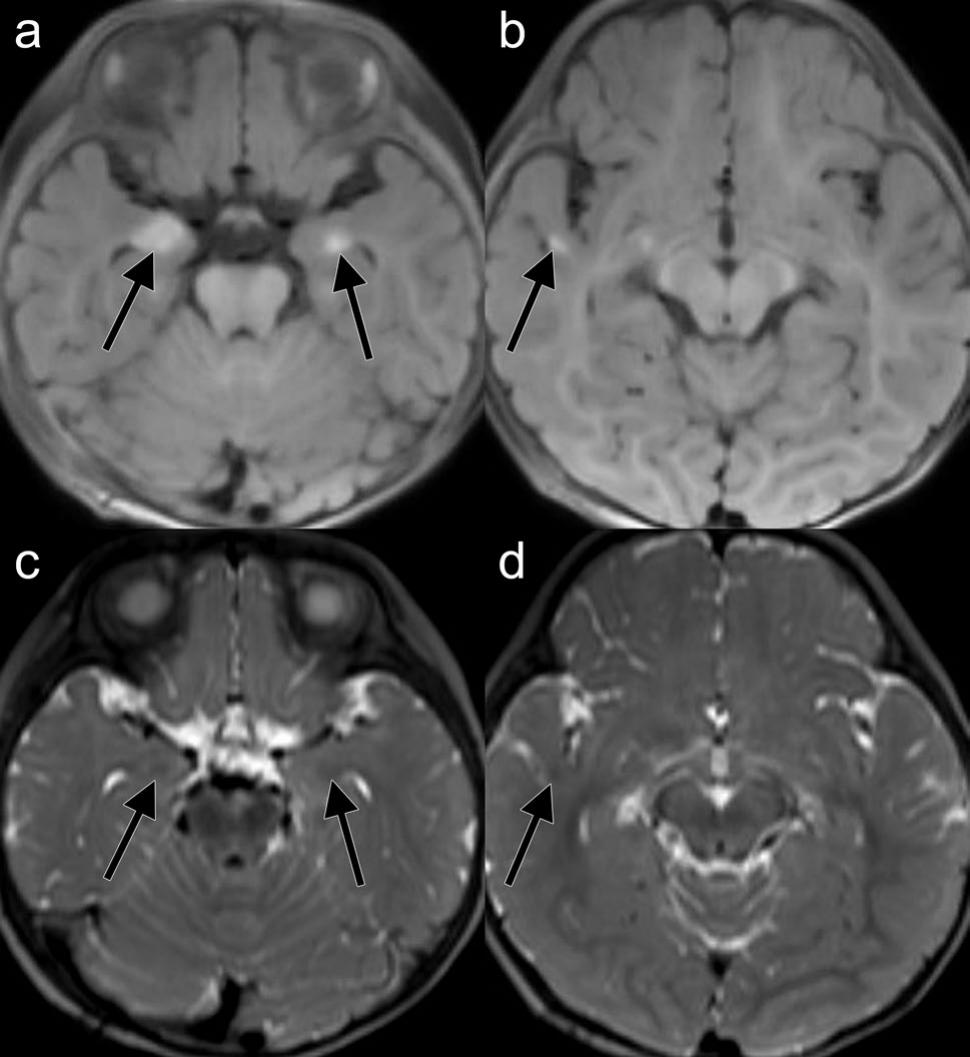
A 4-month-old male with NCM. Hyperintensity lesions on T1WI are present in bilateral amygdalas (**a**) and right temporal cortex (**b**). T2WI shows slightly low signal intensities in the corresponding lesions (**c,d**).

**Figure 3:**
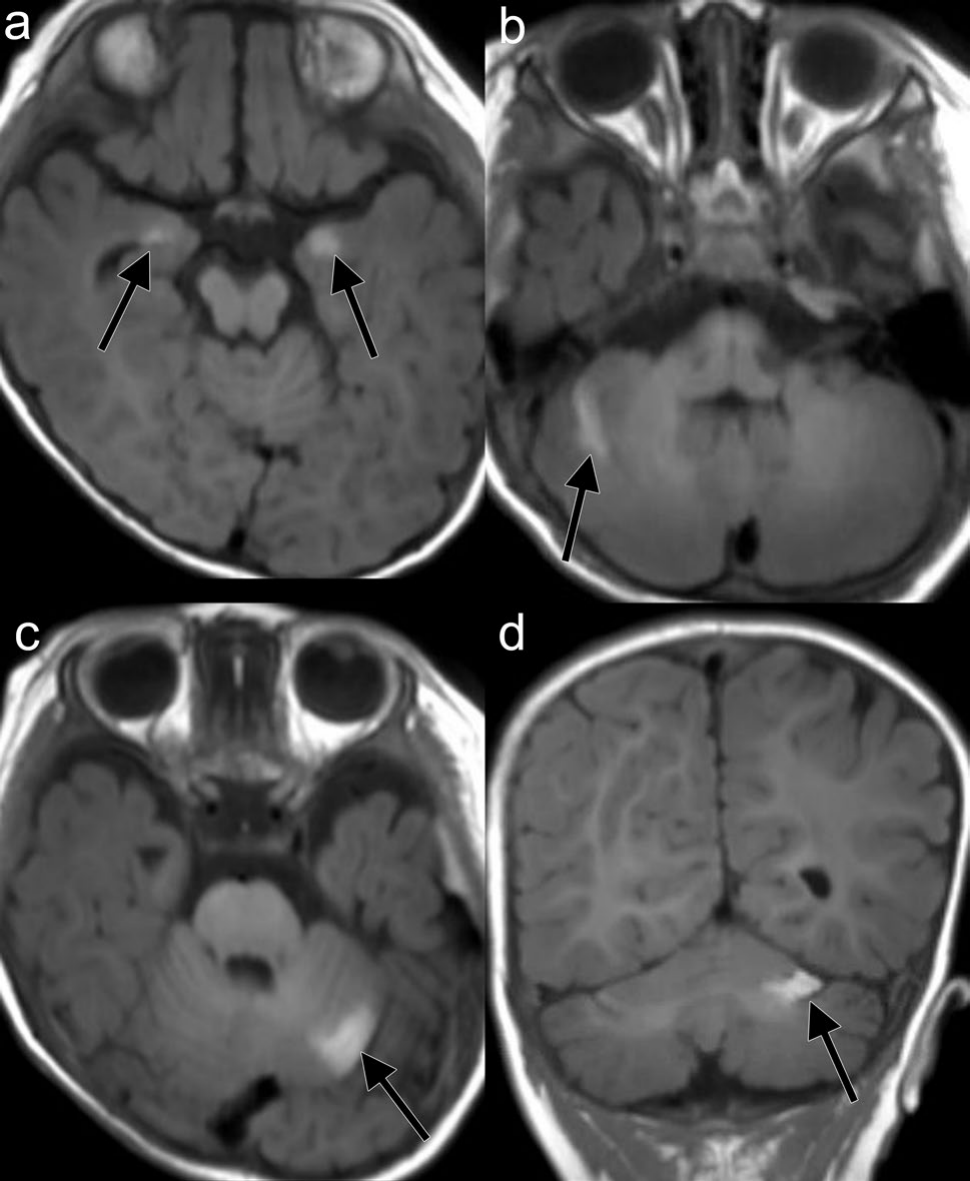
A 4-month-old male with NCM. Hyperintensity lesions on T1WI are present in bilateral amygdalas (**a**) and bilateral cerebellar cortices (**b–d**).

Brain MRI was performed for 38 patients. Other than hyperintensity on T1WI, no patients showed brain abnormalities such as hydrocephalus, Chiari malformation, or syringomyelia. Representative cases of NCM are shown in Figs. 2 and 3.

### Analysis 1

NCM was found in 31.6% (95% confidence interval [CI] 19.0–47.0%) (no previous treatment group: 12/38; 7 males, 5 females). These lesions were most frequently seen in the amygdala, followed by the cerebellum, brainstem, and cerebral hemispheres (Table 1).

**Table 1:** Characteristics of all patients with NCM.

Patient	Age (months)	Sex	Location of NCM	Neurological manifestation	CMN			Included analysis
					Distribution	Size	Satellite nevi	
1	3	F	Lt. amygdala	No	Bathing trunk	G2	3	1, 2
2	4	M	Bil. amygdalas, bil. cerebellum, pons, medulla oblongata, cerebral cortices	No	Bolero	G1	3	1
3	4	F	Multiple brain cortices	No	Bathing trunk	G2	2	1
4	4	F	rt. amygdala, pons, lt. temporal cortex	No	Bolero	G1	3	1, 2
5	4	M	Bil. amygdalas, bil. temporal lobes, rt. cerebellum	No	Back	G1	1	1, 2
6	5	M	Bil. amygdalas, cerebellum	No	Bolero	G2	3	1, 2
7	6	M	Bil. amygdalas	No	Bolero	G2	3	1, 2
8	7	F	Rt. amygdala	No	Bolero	G1	2	1, 2
9	7	M	Lt. amygdala, pons, midbrain, cerebellum	No	Bathing trunk	G2	3	1, 2
10	10	M	Lt. amygdala	No	Bolero	G2	3	1
11	11	M	Rt. Amygdala	No	Bonce	M2	1	1, 2
12	12	F	Bil. amygdalas, rt. temporal lobe	Epileptic seizure	Bathing trunk	G2	3	1

The relationships between characteristics of CMN and NCM are shown in Table 2. Significant association with the presence of NCM were shown by Fisher’s exact test for distribution of CMN (p = 0.002), size of CMN (p = 0.01), number of satellite nevi (p = 0.002), rugosity (p = 0.009) and nodules (p = 0.006).

**Table 2:** Relationship between characteristics of NCM and CMN.

Size*	NCM (+)	NCM (−)	Color	NCM (+)	NCM (−)
M2	1	10	0	4	12
L1	0	4	1	6	7
G1	3	8	2	2	7
G2	8	4	Total	12	26
Total	12	26			

Distribution**	NCM (+)	NCM (−)	Rugosity****	NCM (+)	NCM (−)
Back	1	6	0	1	13
Bathing trunk	4	0	1	6	11
Bolero	6	7	2	5	2
Bonce	1	13	Total	12	26
Total	12	26			

Satellite nevi***	NCM ( +)	NCM (−)	Nodule*****	NCM ( +)	NCM (−)
0	0	4	0	2	18
1	2	13	1	9	7
2	2	7	2	1	1
3	8	2	Total	12	26
Total	12	26			

The size of the CMN was categorized by calculating the projected adult size as follows: G2, > 60 cm; G1, 40–60 cm; L2, 30–40 cm; L1, 20–30 cm; M2, 10–20 cm; and M1, 1.5–10 cm. The number of satellite nevi was categorized into grades 0–3 as follows: grade 0, no nevi; grade 1, 1–19 nevi; grade 2, 20–50 nevi; and grade 3, > 50 nevi.

Colour: grade 0, none; grade 1, moderate; grade 2, hyperheterogeneity.

Rugosity: grade 0, none; grade 1, moderate; grade 2, marked.

Nodules: grade 0, none; grade 1, scattered; grade 2, extensive dermal or subcutaneous.

*p = 0.01; **p = 0.002; ***p = 0.002; ****p = 0.009; *****p = 0.006.

### CMN

The relationship between the distribution of CMN and the presence of NCM is shown in Table 3. In terms of anatomical localization, the most frequent sites of CMN were the scalp and back, followed by the flank. These localizations of CMN overlapped in most patients. No patients were categorized into “multiple CMN” or showed CMN on the hands, feet or lower legs.

**Table 3:** Distribution of CMN and presence of NCM.

Localization of CMN	n	NCM		Sex	
		NCM ( +)	NCM (−)	Male	Female
Face	9	1	8	4	5
Scalp	19	6	13	10	9
Neck	8	5	3	4	4
Shoulder	11	5	6	7	4
Upper back	14	5	9	9	5
Middle back	16	7	9	12	4
Lower back	13	6	7	8	5
Breast	8	4	4	5	3
Abdomen	7	5	2	4	3
Flank	13	6	7	8	5
Gluteal	7	4	3	2	5
Genital	4	4	0	1	3
Upper arm	9	4	5	5	4
Forearm	2	2	0	1	1
Thigh	3	3	0	1	2

### Analysis 2

NCM was found in 25.0% (95%CI 13.0–42.0%) (no previous MRI examination and treatment group: 8/32; 5 males, 3 females) of all patients in our cohort study.

## Discussion

In this study, NCM was found in 31.6% (Analysis 1, 12/38) and 25.0% (Analysis 2, 8/32, no previous MRI examination and treatment group) of patients with CMN. The incidence of large CMN has traditionally been estimated as around one in 20,000–50,000 newborns^3,5,22,23^. The incidence of NCM among patients with CMN has varied between studies because of differences in study designs and the rarity of the pathology^3,5,22,23^. The accurate incidence of NCM among patients with CMN outside western countries remains unclear, since only a few studies have been published to date^24,25^. The evidence accumulated in the present study may contribute, at least among Japanese populations, to alleviating some of the anxiety of patients, families, and physicians before patients undergo brain MRI. The distribution of NCM lesions in this study, particularly the predominance of the amygdala, was consistent with findings from previous studies^3,7,11,14–16,26–28^.

Distribution, size and characteristic features (rugosity and nodules) of CMN and the number of satellite nevi were strongly associated with the presence of NCM. Among these, a tendency was seen for NCM to show a “bathing trunk”-type distribution, large-sized CMN and larger number of satellite nevi. CMN on the posterior axis (paraspinal, head, and neck regions) and the presence of a greater number of satellite nevi have appeared to represent the strongest risk factors for NCM in past studies^12–14,29,30^. The relationship between characteristics of CMN and the incidence of NCM should be clarified in further studies with larger cohorts.

Whether all patients with CMN should undergo brain MRI remains an open question. A positive result on MRI for NCM may impact decisions regarding timing and extent of surgical management for CMN, because brain MRI can suggest whether NCM is stable or progressive and indications for aggressive treatment of CMN will be considered based on the prognosis of NCM^31^. Patients with NCM may be asymptomatic, but occasionally present with headache associated with hydrocephalus, seizure, mental retardation, and various abnormalities of the CNS, including Dandy–Walker syndrome, Chiari malformation, syringomyelia, and lissencephaly^7,15,32^. Brain MRI is currently the most appropriate modality to screen for coexistence of these abnormalities. However, some studies have cast doubt on the effectiveness of routine MRI itself as a screening test for NCM, particularly in patients who are neurologically asymptomatic^22,24^.

CMN are present at birth and have developed in utero in the absence of ultraviolet exposure. CMN is caused by somatic mosaicism in melanocytic precursors, and NRAS mutations can be found in 80% to 95% of CMNs^33^, and no BRAF mutation was found^34^. The decision for resection of CMN is complicating due to many factors, including family wishes, size and location of CMN, patient age, general health, and prognosis^17^. Intralesional injection of proinflammatory squaric acid dibutylester (SADBE) reportedly achieved major regressions of CMN in mice^35^. Although still in preclinical state, such topical approach may be an alternative of surgical resection.

The ectoderm cells destined to generate neural crest precursor cells undergo an epithelial-to-mesenchymal conversion and then migrate to diverse sites in the embryo where they differentiate into multiple derivatives such as neurons and melanocytes of the skin^36^. The epidermis represents the outermost layer of the skin, which is predominantly formed by proliferating and differentiating keratinocytes, and the epidermis also contains melanocytes, Merkel cells, and Langerhans cells. Of note, the skin constituents communicate cross-functionally through local nervous, immune, and endocrine systems to maintain essential epidermal functions^37,38^.

Several limitations should be noted in this study. First, the number of patients with CMN was limited. Although our institute is a high-volume center for patients with CMN in Japan, the relatively short enrollment period and inherent rarity of this pathology would have contributed to limiting the number of patients enrolled in this study. Further registry studies are expected in this area. Second, the number of patients with NCM itself was also quite limited. However, the evidence related to the incidence of NCM confirmed from brain MRI in this prospective cohort is valuable despite the small cohort. Third, particularly among infants, accurate evaluation of neurological and clinical manifestations is often difficult. Some neurologically symptomatic patients may have been overlooked in this study. Fourth, the prognosis of NCM was not evaluated in this study.

In conclusion, NCM was found in 31.6% of patients with CMN in this Japanese cohort, and in 25.0% of patients with no prior examination or treatment. The distribution, size and particular characteristic features (rugosity and nodules) of CMN and number of satellite nevi appear strongly associated with the existence of NCM. The results accumulated in the present study may guide a future registry study with a large cohort of CMN patients.

## Data Availability

The data of this study are available from the corresponding author upon reasonable request.
